# Metabolic Alterations in a *Drosophila* Model of Parkinson’s Disease Based on *DJ-1* Deficiency

**DOI:** 10.3390/cells11030331

**Published:** 2022-01-20

**Authors:** Cristina Solana-Manrique, Francisco José Sanz, Isabel Torregrosa, Martina Palomino-Schätzlein, Carolina Hernández-Oliver, Antonio Pineda-Lucena, Nuria Paricio

**Affiliations:** 1 Departamento de Genética, Facultad CC Biológicas, Instituto Universitario de Biotecnología y Biomedicina (BIOTECMED), Universidad de Valencia, 46100 Burjassot, Spain; crisoman@alumni.uv.es (C.S.-M.); fco.sanz@uv.es (F.J.S.); itoblas@alumni.uv.es (I.T.); 2 Centro de Investigación Príncipe Felipe, 46012 Valencia, Spain; mpalomino@cipf.es; 3 Instituto de Investigación Sanitaria La Fe, Hospital Universitario y Politécnico La Fe, 46026 Valencia, Spain; chernandez@cnio.es (C.H.-O.); apinedal@unav.es (A.P.-L.); 4 Programa de Terapias Moleculares, Centro de Investigación Médica Aplicada, Universidad de Navarra, 31008 Pamplona, Spain

**Keywords:** *Drosophila*, *DJ-1*, Parkinson’s disease, metabolomics, NMR spectroscopy

## Abstract

Parkinson’s disease (PD) is the second-most common neurodegenerative disorder, whose physiopathology is still unclear. Moreover, there is an urgent need to discover new biomarkers and therapeutic targets to facilitate its diagnosis and treatment. Previous studies performed in PD models and samples from PD patients already demonstrated that metabolic alterations are associated with this disease. In this context, the aim of this study is to provide a better understanding of metabolic disturbances underlying PD pathogenesis. To achieve this goal, we used a *Drosophila* PD model based on inactivation of the *DJ-1β* gene (ortholog of human *DJ-1*). Metabolomic analyses were performed in 1-day-old and 15-day-old *DJ-1β* mutants and control flies using ^1^H nuclear magnetic resonance spectroscopy, combined with expression and enzymatic activity assays of proteins implicated in altered pathways. Our results showed that the PD model flies exhibited protein metabolism alterations, a shift fromthe tricarboxylic acid cycle to glycolytic pathway to obtain ATP, together with an increase in the expression of some urea cycle enzymes. Thus, these metabolic changes could contribute to PD pathogenesis and might constitute possible therapeutic targets and/or biomarkers for this disease.

## 1. Introduction

Over the last 25 years, neurodegenerative diseases (NDs) have become a significant challenge to general health, especially in older population groups, and are expected to grow in the future due to an increase in life expectancy [1]. Among them, Parkinson’s disease (PD) is the most common motor disorder, affecting more than 1% of the population over 60 years. PD is caused by the selective and progressive loss of dopaminergic (DA) neurons in the substantia nigra pars compacta (SNpc), which results in striatal dopamine deficiency and leads to disturbances in motor, autonomic and psychiatric functions [2,3,4]. DA neuron loss is accompanied, in some cases, by the presence of α-synuclein aggregates in the surviving neurons, known as Lewy bodies [2,4]. The exact mechanism behind DA neurodegeneration in PD is still unclear, most likely a combination of genetic predisposition and environmental factors [2,5]. Indeed, multiple pathways and mechanisms seem to participate in PD pathogenesis, such as the accumulation of misfolded protein aggregates, mitochondrial dysfunction, increased oxidative stress (OS), energy failure, neuroinflammation, and genetic mutations [6]. Interestingly, recent studies have shown that bioenergetic alterations may play a key role in PD neuropathology. Concretely, an increase in the glycolytic rate was observed in PD models, suggesting that there is a link between glucose metabolism, cellular bioenergetics, redox homeostasis and neuronal death [7,8].

Most PD cases are idiopathic (iPD), whose etiology is multifactorial. However, 5–10% of PD patients suffer from monogenic forms of the disease caused by highly penetrant mutations that are family-linked [2,5]. In recent years, mutations in several genes were associated with these familial forms of PD (fPD), allowing the discovery of different mechanisms underlying PD pathogenesis [5,9]. Indeed, some of the proteins encoded by these genes are involved in a set of molecular pathways that upon perturbation trigger a neuropathology that resembles or is clinically indistinguishable from iPD, except for the age at onset [10]. Among them, *DJ-1* is a causative gene for fPD [11] that was initially described as an oncogene. Nevertheless, other functions were ascribed to the DJ-1 protein such as transcriptional regulation, chaperone and protease activity, scavenger of reactive oxygen species (ROS) or mitochondrial homeostasis [12,13,14]. Remarkably, an over-oxidized and inactive form of the DJ-1 protein was found in brains of iPD individuals, suggesting that it may play a central role in the development of the disease [15]. Therefore, results obtained in PD models with impaired DJ-1 function could be also applicable to human iPD forms.

Currently, PD diagnosis is limited and based on the detection of classic motor symptoms that appear when about 60–80% of SNpc DA neurons are lost, after many years of ongoing disease [16]. Thus, it is an urgent need to find simple, useful and low-cost biomarkers for an early PD diagnosis, in order to address its progression in the initial stages of the disease [17]. Biomarkers are also needed for distinguishing different PD types, predicting the course of the disease, or monitoring the effect of disease-modifying therapies [18,19]. Metabolomic approaches allow the analysis of a great number of low-molecular-weight molecules, offering an overview of the molecular complexity of a biological system and the metabolic pathways that can be altered in a pathological state [20,21]. Therefore, metabolomic studies are being carried out to identify new biomarkers in PD and other NDs [22,23], as well as therapeutic targets and metabolic alterations implicated in PD pathogenesis [23].

*Drosophila melanogaster* has emerged as an important model organism in the study of PD physiopathology. In this scenario, several genetic-based and chemically induced PD models were developed in *Drosophila* [24,25]. Among them, flies harboring mutations in *DJ-1β* (ortholog of *DJ-1* human gene) present typical PD phenotypes, such as motor impairment, and increased levels of OS markers [26,27,28]. In addition, *DJ-1β* mutants exhibit an increased activity of key glycolytic enzymes [7]. Given the great complexity and heterogeneity in the metabolome of PD human samples, as well as their limited availability, models in simpler organisms such as *Drosophila* could be very useful for studying PD-associated, metabolome-wide alterations [29,30,31]. In fact, metabolomic studies were conducted in different fly models of other NDs. For instance, a metabolomic analysis of a *Drosophila* model of Charcot-Marie-Tooth disease based on *GDAP1* deficiency allowed the involvement of insulin signaling in the characteristic neuromuscular degeneration of this disease to be identified [32]. Moreover, a transgenic *Drosophila* model of Huntington’s disease was used to discover metabolic perturbations at two stages of the disease [33]. Regarding PD, Shukla et al. [21] performed a metabolomic analysis in a paraquat-induced *Drosophila* model of iPD, in which altered levels of amino acids, lipids and carbohydrates were identified [21]. In such a scenario, we aimed to find new pathways underlying PD pathogenesis and/or new potential biomarkers by carrying out metabolomic analyses in *DJ-1β* mutants and control flies at different ages. Changes in metabolite levels in PD model flies compared to controls led us to identify alterations in amino acids metabolism, a switch from the tricarboxylic acid (TCA) cycle to glycolysis, as well as disturbances in other pathways such as the urea cycle (UC). We discuss the relevance of the detected metabolic alterations in the context of this disease.

## 2. Materials and Methods

### 2.1. Drosophila Strains

All stocks and crosses were cultured on standard *Drosophila* food at 25 °C. For this work, *DJ-1β^ex54^* mutant flies (hereafter called *DJ-1β*) were used as PD model flies [34] and *y^1^,w^1118^* flies were used as controls.

### 2.2. Metabolite Extraction

Metabolite extraction was performed as previously described in [32]. Twelve samples of 1-day-old and 15-day-old individuals were prepared for each batch of *DJ-1β* mutants and controls, containing fifteen female flies each. In line with our previous studies, we used female flies in all experiments [7,26,27,28,35,36,37]. They were frozen in a microtube by immersion in liquid nitrogen. Then, 240 µL of ice-cold methanol and 48 µL of ice-cold Milli-Q water were added to each vial. After 5 min, flies were homogenized with a small mortar for 60 s. Samples were vortexed and 120 µL of ice-cold CHCl_3_ and 120 µL of ice-cold Milli-Q water added and vortexed again. After 15 min at 4 °C, samples were centrifuged at 10,000× *g* during 15 min at 4 °C. The resulting two phases (upper phase: polar metabolites, lower phase: non-polar metabolites) were separated. Solvents from the polar phase were eliminated by freeze–thawing, and samples were stored at −80 °C until measurement.

### 2.3. NMR Analysis

Metabolite extracts were allowed to thaw for 5 min at 4 °C and dissolved in 550 µL of NMR buffer (0.1 M phosphate buffer pH 7.4 in D2O, with 0.1 mM 3-(Trimethylsilyl)propionic-2,2,3,3-d4 acid sodium salt (TSP) as internal standard) for the polar phase. Polar samples were analyzed on a 600 MHz Bruker NMR spectrometer (Bruker, MA, USA) equipped with a cryoprobe using a 1D NOESY experiment, including presaturation for water signal suppression and a 50 ms mixing delay. Spectra were acquired with 512 scans, a relaxation delay of 4 s and a spectral width of 18,028 Hz and processed with an exponential line broadening factor of 0.5. For metabolite identification, 2D TOCSY and HSQC experiments were acquired for selected samples. All experiments were performed at 37 °C.

### 2.4. Metabolite Assignment and Quantification

NMR spectra were processed and analyzed with NmrProcflow (NMRProcFlow, Bordeaux, France) [38]. Baseline correction was performed with local level 5 and spectra alignment with the option “least square”. Bucketing was applied manually with a SNR of 3, resulting in 310 buckets. The signals corresponding to methanol, chloroform, TSP and H_2_O were excluded. Data were exported from NMRProcFlow tool and integration values were normalized to total intensity (CSN) to minimize variability. For the statistical analysis, a multivariate data analysis was performed using SIMCAP 12.0 Software (Umetrics, Umeå, Sweden) with the normalized integral values. First, spectra were normalized with Probabilistic Quotient Normalization. Principal component analysis (PCA) was performed for a first overview to evaluate clustering trends between samples and to identify outliers. Secondly, Orthogonal Partial Least Squares Discriminant Analysis (OPLS-DA) was performed for discrimination analysis between groups. Furthermore, the analysis of S-plots allowed the metabolites to be defined that were essential for forming the discrimination between groups. OPLS-DA models were validated by permutation. In a second step, a pathway analysis was applied using the web-based software for metabolomics, MetaboAnalyst 5.0 Software (https://metaboanalyst.ca/; accessed on 3 March 2021).

### 2.5. Measurement of ATP Levels

ATP levels were measured using the ATP Determination Kit (Invitrogen, Waltham, MA, USA) following manufacturer’s instructions. Briefly, groups of five 15-day-old *DJ-1β* mutant and control female flies were homogenized in 200 µL of reaction buffer (supplied by the commercial kit). Then, fly extracts were boiled for 4 min and centrifuged at 18,500× *g* for 10 min at 4 °C in order to discard debris. Subsequently, 5 µL of fly extracts were added to 100 µL of the standard reaction solution in a white 96-well plate and luminescence was measured using an Infinite 200 PRO reader (Tecan, Männedorf, Switzerland). All experiments were performed in triplicate and the results are expressed as relative luminescence intensity per mg of protein normalized to control flies.

### 2.6. Enzymatic Activity Assays

Protein extracts were obtained from groups of twenty *DJ-1β* mutant and control female flies, as described in [7]. Aconitase (Aco; EC 4.2.1.3) activity was measured with the Aconitase Activity Assay Kit (#MAK051; Sigma-Aldrich, St. Louis, MO, USA), following manufacturer’s instructions, and assays were performed in triplicate. Succinate dehydrogenase (SDH; EC 1.3.5.1) activity was measured using a protocol adapted from [39]. First, mitochondrial extracts were obtained from groups of 30 *DJ-1β* mutant and control female flies, as described in [40]. Then, 50 µL of mitochondrial extracts were added to 50 µL of assay buffer (4 mM sodium azide, 50 µM 2,6-dichlorophenolindophenol and 2 µg/mL rotenone) and transferred to a 96-well plate. The reaction was started by adding succinate to a final concentration of 10 mM. Absorbance was measured at 600 nm using an Infinite 200 PRO reader (Tecan) every 30 s for 20 min at 25 °C. Sample absorbance levels were measured, subtracting their corresponding blanks. Assays were performed in triplicate.

### 2.7. RT-qPCR Analyses

Total RNA from groups of ten 15-day-old *DJ-1β* mutant or control female flies was extracted and reverse transcribed as described in [7]. RT-qPCR reactions were performed as in [7], and the following pairs of primers were used: *tubulin* direct primer (5′-GATTACCGCCTCTCTGCGAT-3′); *tubulin* reverse primer (5′-ACCAGAGGGAAGTGAATACGTG-3′); *arg* direct primer (5′-AGCTTTGACATCGACGCCTT-3′); *arg* reverse primer (5′-CTCCACGATGCTGATTCCCT-3′); *argL* direct primer (5′-CGCATCACATTATCGGTCGC-3′); and *argL* reverse primer (5′-TCTCCCAGCGGCAAATCAG-3′).

### 2.8. Statistical Analysis and Data Representation

Data are expressed as means ± standard deviation (s.d.). The significance of differences between means was assessed using a t-test except for NMR analysis where a different statistical analysis was performed (see section NMR analysis). Differences were considered significant when * *p* < 0.05. Data representations were performed using GraphPad Prims 6.0 Software (GraphPad Software, Inc., Chicago, IL, USA).

## 3. Results

### 3.1. Impact of DJ-1β Loss on the General Metabolic Profile

Previous studies showed that a lack of *DJ-1* function leads to several metabolic alterations [7,41,42,43]. Indeed, we recently demonstrated that *DJ-1β* mutant flies and *DJ-1*-deficient human neuroblastoma cells showed an increase in the glycolytic pathway [7]. In order to identify additional metabolic changes caused by the loss of *DJ-1β* function that could contribute to PD pathophysiology, we undertook metabolomic analyses in *DJ-1β* mutants and control flies by NMR spectroscopy. These were performed in 1-day-old and 15-day-old flies to detect early alterations that may constitute promising biomarkers for PD diagnosis, as well as later biomarkers that could help to evaluate disease progression.

**Figure 1:**
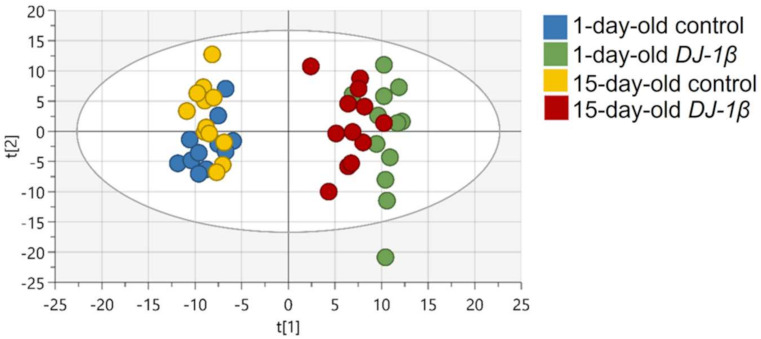
PCA-X score plots for all four experimental groups: 1-day-old control flies (blue), 1-day-old *DJ-1β* mutant flies (green), 15-day-old control flies (yellow) and 15-day-old *DJ-1β* mutant flies (red).

First, spectral peaks were obtained in all four experimental groups, followed by the metabolite assignment of peaks that were statistically relevant. After this, the relative abundance of each metabolite in *DJ-1β* mutants and control flies at both ages was established, in order to evaluate the differences between these groups. A multivariable statistical analysis was performed to identify the most significant changes in the metabolomic assays, which are shown in Tables S1–S3. Metabolic alterations were analyzed by PCA to identify outliers and to evaluate the existence of any trend or aggrupation due to another variable. As shown in Figure 1 all samples grouped according to the phenotype and the specific age, especially in *DJ-1β* mutant flies for which both ages are distinctly separated. This evaluation was followed by an OPLS-DA to establish the models that allow the differentiation of metabolic changes for all four experimental groups. In all comparisons, optimal models were obtained with R^2^ values close to 1 and reasonable predictive values (Q^2^) (data not shown).

**Figure 2:**
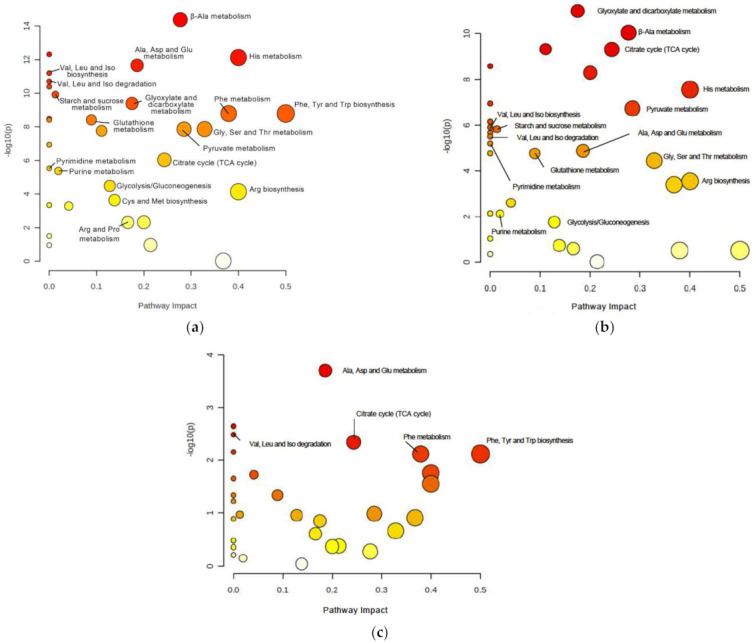
Pathway analysis reveals metabolic alterations in *DJ-1β* mutant flies. Pathway enrichment analysis of differentially expressed metabolites between (**a**) 1-day-old *DJ-1β* mutant and control flies, (**b**) 15-day-old *DJ-1β* mutant and control flies, and (**c**) 1-day-old and 15-day-old *DJ-1β* mutant flies. Pathway impact represents the importance and number of altered metabolites in a given pathway, also indicated by the size of the corresponding circle. Colors represent the significance of changes in the pathways. Red color indicates higher values of –lop10(*p*) and more significant changes in that pathway.

To identify relevant pathways that could be perturbed in *DJ-1β* mutants at different ages, we used the MetaboAnalyst 5.0 software to examine metabolites showing significant alterations in such flies compared to controls (Tables S4–S6). As shown in Figure 2a,b and Table 1 and Table 2, the most relevant changes with a considerable impact power between *DJ-1β* mutant and control flies at both ages belonged to several amino acid metabolism pathways, the TCA cycle (impact = 0.24), pyruvate metabolism (impact = 0.28) and glycolysis/gluconeogenesis pathways (impact = 0.13). In addition, comparisons between 1-day-old and 15-day-old *DJ-1ß* mutant flies showed possible alterations in alanine, aspartate, and glutamine metabolism (impact = 0.19); phenylalanine, tyrosine, and tryptophan biosynthesis (impact = 0.50); phenylalanine metabolism (impact = 0.38); and TCA cycle pathway (impact = 0.24) (Figure 2c, Table 3). In summary, the most significant metabolic changes found in *DJ-1ß* mutants compared to control flies point to alterations in amino acid levels and their corresponding metabolism, as well as in carbohydrate metabolism. Thus, components of these pathways may constitute therapeutic targets and/or potential biomarkers in PD. These findings represent an initial step to further investigate genes, enzymes, or metabolites implicated in these pathways that contribute to PD physiopathology.

### 3.2. Alterations in Amino Acid Content in DJ-1β Mutant Flies

**Figure 3:**
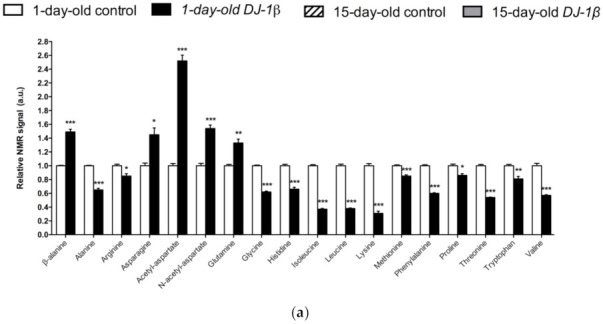

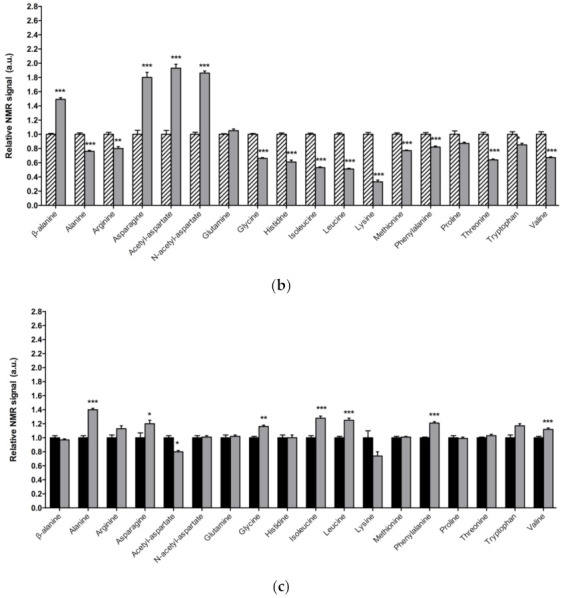
Alterations in amino acid content in PD model flies. Relative NMR signals of amino acids between (**a**) 1-day-old *DJ-1β* mutant and control flies, (**b**) 15-day-old *DJ-1β* mutant and control flies, and (**c**) 1-day-old and 15-day-old *DJ-1β* mutant flies. In all cases, error bars show s.d. from twelve independent samples (*, *p* < 0.05; **, *p* < 0.01; ***, *p* < 0.001).

In Figure 3a, a detailed overview of the differences in amino acids found between 1-day-old *DJ-1β* mutants and control flies is shown. There is a consistent reduction in the amounts of all the amino acids detected in the metabolomic analysis, except for β-alanine, acetyl-aspartate, N-acetyl-aspartate, asparagine, and glutamine, which increase in 1-day-old *DJ-1β* mutants when compared to control flies. Similar results are found when comparing the amino acid profile of 15-day-old *DJ-1β* mutants and control flies with the exception of glutamine, which showed no significant differences between both genotypes (Figure 3b). Among the amino acids whose levels are reduced in PD model flies we found three branched-chain amino acids (BCAAs) (leucine, isoleucine and valine), which promote protein synthesis in the muscle [44], and essential amino acids (histidine, isoleucine, leucine, lysine, methionine, phenylalanine, threonine, tryptophan and valine). Interestingly, a reduction in both BCAAs and essential amino acids was associated with an increase in the clinical severity of PD patients [45]. It is also noteworthy that there is a decrease in tryptophan levels in 1-day-old and 15-day-old *DJ-1β* mutant flies when compared to controls of the same age (Figure 3a,b). Tryptophan is an essential amino acid that participates in the kynurenine pathway to produce NAD^+^, and whose metabolism is altered in PD [46]. A further evaluation of the amino acid content led us to observe an increase in alanine, asparagine, glycine, isoleucine, leucine, phenylalanine and valine levels and a decrease in acetyl-aspartate in 15-day-old compared to 1-day-old *DJ-1β* mutant flies (Figure 3c). These results are consistent with those found in the amino acid profile in serum from patients with early or late PD, where alterations in alanine, arginine, phenylalanine and threonine were detected [47]. Therefore, these amino acids could be relevant in early PD diagnosis.

### 3.3. DJ-1β Deficiency Leads to Changes in Carbohydrate Metabolism

As mentioned above, *DJ-1β* mutant flies show alterations in the TCA cycle, pyruvate metabolism and glycolytic/gluconeogenesis pathways (Figure 2). These results support previous studies in which we demonstrated that PD model flies present an enhancement of glycolysis [7]. Therefore, we decided to delve into the study of carbohydrates metabolism, since several investigations emphasized the importance of glucose usage in PD [48,49].

**Figure 4:**
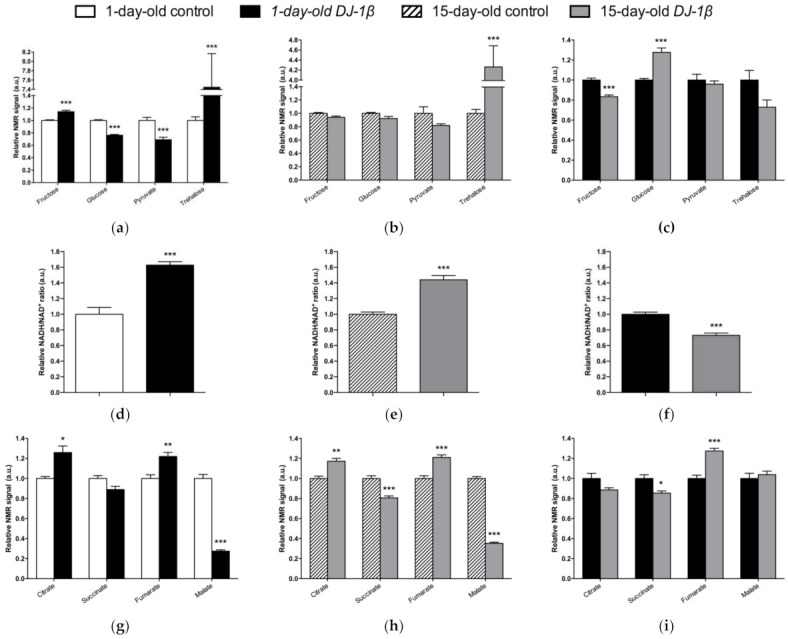
Alterations in carbohydrate content in *DJ-1β* mutant flies. Relative NMR signals of selected carbohydrates in between (**a**) 1-day-old *DJ-1β* mutant and control flies, (**b**) 15-day-old *DJ-1β* mutant and control flies, and (**c**) 1-day-old and 15-day-old *DJ-1β* mutant flies. Relative NADH/NAD^+^ ratio in (**d**) 1-day-old *DJ-1β* mutant and control flies, (**e**) 15-day-old *DJ-1β* mutant and control flies, and (**f**) 1-day-old and 15-day-old *DJ-1β* mutant flies. Relative NMR signals of TCA cycle intermediates comparing (**g**) 1-day-old *DJ-1β* mutant and control flies, (**h**) 15-day-old *DJ-1β* mutant and control flies, and (**i**) 1-day-old and 15-day-old *DJ-1β* mutant flies. In all cases, error bars show s.d. from twelve independent samples (*, *p* < 0.05; **, *p* < 0.01; ***, *p* < 0.001).

Regarding soluble sugars detected in the metabolic profiles, we found that 1-day-old *DJ-1β* mutants exhibit a decrease in glucose and pyruvate levels, and an increase in fructose and trehalose when compared to control flies of the same age (Figure 4a). An increase in trehalose was also observed in 15-day-old PD model flies, but changes in other sugars were not detected at that age, despite a decreasing trend in glucose and pyruvate levels being observed (Figure 4b). When comparing soluble sugar levels between 1-day-old and 15-day-old *DJ-1β* mutant flies, a reduction in fructose and an increase in glucose levels are observed in 15-day-old flies (Figure 4c), which could imply that glucose metabolism is reduced at late stages of PD. Taken together, our results from metabolomic analyses suggest that there is a modification in the usage of soluble sugars as an energy source to produce ATP.

### 3.4. Switch from TCA Cycle to Glycolysis in DJ-1β Mutant Flies

Glycolysis converts glucose to pyruvate, which constitutes an important bridge between this pathway and the mitochondrial TCA cycle to produce high amounts of ATP through the electron transport chain (ETC) [50]. In this scenario, NADH/NAD^+^ plays a crucial role in metabolism and highlights the main route to obtain ATP in cells [51]. As shown in Figure 4d,e, both 1-day-old and 15-day-old PD model flies show increased NADH/NAD^+^ ratio when compared to control flies of the same age. This increase is higher in 1-day-old *DJ-1β* mutants (Figure 4f). These results, together with other studies conducted by our group [7], suggest that there is a switch from the TCA cycle to glycolysis to obtain ATP. According to this, we observed changes in the NMR signals of TCA cycle intermediates between the PD model and control flies (Figure 4g–i). Specifically, 1-day-old and 15-day-old *DJ-1β* mutant flies showed an increase in citrate and fumarate and a decrease in malate levels when compared to control flies of the same age (Figure 4g,h). The increase in fumarate levels was higher in 15-day-old *DJ-1β* mutants (Figure 4i). In addition, 15-day-old PD model flies had decreased succinate levels when compared to controls (Figure 4h).

**Figure 5:**
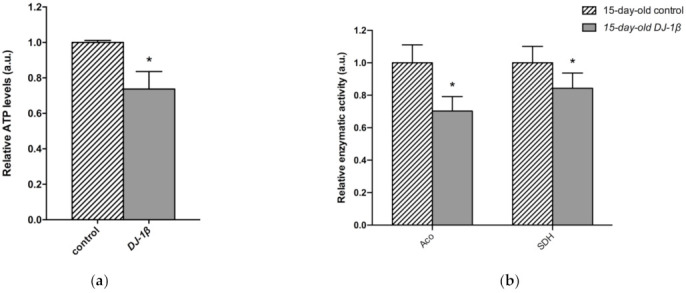
Metabolic switch to glycolysis in 15-day-old *DJ-1β* mutant flies. (**a**) Relative ATP levels in 15-day-old *DJ-1β* mutant flies compared to controls of the same age. (**b**) Relative enzymatic activity of aconitase (Aco) and succinate dehydrogenase (SDH) from the TCA cycle in 15-day-old *DJ-1β* mutant flies compared to controls. In all cases, error bars show s.d. from three independent experiments, in which three biological replicates were used (*, *p* < 0.05).

As changes in TCA cycle metabolites were more evident in 15-day-old *DJ-1β* mutants, we decided to focus our studies on this experimental group. We hypothesized that mitochondria became less efficient when producing ATP in 15-day-old PD model flies, which was confirmed by measuring ATP levels. As expected, they were reduced in *DJ-1β* mutants when compared to control flies (Figure 5a). This could be explained by an alteration in the TCA cycle. On the one hand, citrate is an essential TCA cycle intermediate that acts as a substrate of the Aco enzyme. Aco catalyzes the stereospecific isomerization of citrate to isocitrate, and acts as a biosensor of ROS and iron [52]. We observed a decrease in Aco activity in 15-day-old *DJ-1β* mutants compared to control flies (Figure 5b), which may explain the reduction in citrate levels obtained in the metabolomic analysis (Figure 4g,h). On the other hand, increased fumarate, and decreased succinate levels in 15-day-old *DJ-1β* mutants compared to controls could be explained by changes in SDH activity in PD model flies. Mitochondrial SDH oxidizes succinate to fumarate in TCA cycle and reduces ubiquinone in ETC, linking both routes [53,54]. To confirm this assumption, we measured SDH activity in 15-day-old *DJ-1β* mutants. Our results showed that it was decreased when compared to control flies (Figure 5b), therefore suggesting that fumarate must be produced by other ways. In summary, we demonstrated that PD model flies show a shift in metabolic pathway selection to glycolysis due to impairment in the TCA cycle.

**Figure 6:**
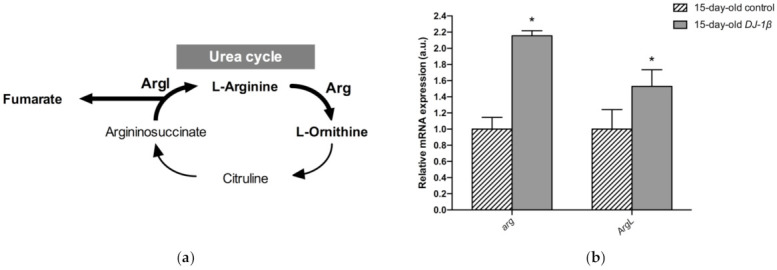
Alterations in the urea cycle in 15-day-old *DJ-1β* mutant flies. (**a**) Schematic diagram of the urea cycle. Fumarate is a final metabolite that can enter the TCA cycle. (**b**) Expression levels of selected enzymes of the urea cycle, arginase (*Arg)* and argininosuccinate lyase (*ArgL*). Error bars show s.d. from four independent experiments (*, *p* < 0.05).

Another possible explanation for the increase in fumarate levels found in 15-day-old *DJ-1β* mutants compared to controls could be an alteration in the UC. Fumarate acts as a bridge between the TCA cycle and the UC. It is one of the final UC products, and is transported into the mitochondria where it can be used as a substrate of the TCA cycle (Figure 6a) [55,56]. Our metabolomic analyses showed an increase in fumarate levels but also a reduction in arginine levels in 15-day-old *DJ-1β* mutants. To determine whether these results could reflect the existence of changes in the UC in 15-day-old PD model flies, we analyzed the expression of the genes encoding the enzymes arginase (*arg*, EC 3.5.3.1) and argininosuccinate lyase (*Argl*, EC 4.3.2.1) (Figure 6a). Arg is a metalloenzyme that catalyzes the synthesis of L-ornithine from L-arginine, generating urea [56], while Argl catalyzes the reversible conversion of argininosuccinate into L-arginine and fumarate [57]. RT-qPCR analyses revealed that the expressions of both genes (*arg* and *Argl*) were increased in *DJ-1β* mutants compared to control flies (Figure 6b). These results are in agreement with changes in fumarate and arginine levels observed in PD model flies and suggest that alterations in the UC could be relevant for PD physiopathology. Further experiments will be required to confirm this assumption.

## 4. Discussion

PD is an incurable and complex disease in which the mechanisms that cause DA neuronal death are not yet clear [2,5]. Some authors described a link between redox imbalance, energy failure and metabolic disturbances in PD, leading to the idea that it might be considered a metabolic disease [7,8]. In this study, we used NMR spectroscopy for metabolomic profiling in *DJ-1β* mutants and control flies of different ages. The aim of this study was to detect metabolic alterations that might be relevant in PD physiopathology, as well as identify potential therapeutic targets and biomarkers. To date, several metabolomic analyses were performed in the plasma, cerebrospinal fluid or blood of PD patients [47,58,59,60,61,62,63]. However, human samples are limited to specific tissues and do not allow for a complete examination of the disease [29,30,31]. To circumvent this limitation, we analyzed metabolites in the whole body of PD model flies. This experimental approach allows a complete panorama of PD-associated metabolic defects to be obtained, which are not restricted to the brain and can influence the physiology of the entire fly [33]. To date, only one similar study has been performed in another *Drosophila* PDf model based on *PINK1* deficiency [64]. Our results confirm that several metabolic pathways, such as amino acid catabolism/anabolism, glycolysis, the TCA cycle, or the UC cycle, might be altered in PD.

Neurons require high levels of energy, which is mainly produced by mitochondria. However, mitochondria become less efficient with age as well as in several NDs, such as PD, thus leading to a reduction in ATP levels, while ROS production increases [50]. In this scenario, amino acids play an important role in the brain, since some of them can be metabolized to fuel cellular energetics when there is insufficient energy [56]. The amino acid profile observed in 1-day-old and 15-day-old *DJ-1β* mutants is consistent with amino acids anabolism/catabolism imbalance in PD model flies, especially in younger flies (Figure 3). Interestingly, an increase in amino acid catabolism, through transformation to pyruvate and acetyl-CoA or other TCA cycle intermediates to produce energy [65], could compensate ATP deficit due to impaired mitochondrial function. Moreover, the reduction in amino acid levels could be also consistent with impairment in protein synthesis in muscles, which could exacerbate motor symptoms in PD patients [44].

Among the amino acids whose levels are altered in PD model flies compared to controls, we found BCAAs. BCAAs metabolism/degradation is closely related to carbon metabolism, in particular to glycolysis and the TCA cycle, since BCAAs can be transformed to succinyl-CoA that fuels TCA cycle [65]. PD model flies exhibit decreased levels of BCAAs probably due to its degradation to produce energy. A similar situation was found in a metabolomic analysis performed in sebum samples of PD patients [66], thus supporting the validity of the results obtained in our *Drosophila* PD model. Interestingly, a clinical trial (NCT01662414) was performed in PD patients to investigate the effect of whey protein supplementation, an important source of BCAAs. The results showed that this supplementation was able to increase BCAAs and essential amino acid levels, as well as reduced glutathione levels, a key antioxidant that prevents the oxidative damage of DA neurons [45]. In relation to this, a recent study showed that whey protein supplementation improved motor symptoms in PD patients due to an increase in muscle regeneration [67]. Our results indicate that BCAAs metabolism seems to be decreased in 15-day-old compared to 1-day-old *DJ-1β* mutant flies. This decrease, especially in aged flies, could enhance PD-related phenotypes, as shown in PD patients [45]. Therefore, BCAAs metabolism could constitute a possible therapeutic target to investigate new PD treatments. Levels of other proteinogenic amino acids are also reduced in *DJ-1β* mutant flies. For example, they present decreased tryptophan levels, whose metabolism is related to neurodegeneration in PD patients at early stages [46,65,68]. Another proteinogenic amino acid affected in *DJ-1β* mutants is glycine, a small amino acid with neuroprotective effects in neuroinflammation, ROS-related damage, and synaptic dysfunction through JNK signaling inactivation [69]. It is likely that a decrease in glycine levels in *DJ-1β* mutants could be contributing to PD pathology. Moreover, there is a significant change in glycine levels when comparing metabolomes of 1-day-old to 15-day-old mutant flies, thus suggesting that this amino acid could be a possible biomarker of PD progression.

PD model flies also exhibit significant increased levels of some non-proteinogenic amino acids compared to controls such as β-alanine. This result could indicate an increase in pyrimidine degradation, as β-alanine is the final product of this pathway. Similar results were found in *pink1* mutant flies, another *Drosophila* fPD model [64]. Moreover, an increase in β-alanine levels causes taurine depletion, which was related to nerve degeneration [70]. Therefore, alterations in β-alanine metabolism and related pathways could be contributing to PD physiopathology. On the other hand, N-acetyl-aspartate is the most abundant non-proteinogenic amino acid in the central nervous system and a marker of neuronal integrity. It is synthetized in the mitochondria from aspartate and acetyl-CoA, and is then transported to the cytosol, where it is metabolized into aspartate and acetate. Its synthesis increases in absence of ATP due to the use of acetate as a source of energy [71]. We found that PD model flies showed an important increase in N-acetyl-aspartate (Figure 3), which could reflect a deficiency in its metabolism, since acetate levels were also decreased in 15-day-old *DJ-1β* mutants compared to control flies of the same age (Table S2).

Alterations in energy metabolism and glucose uptake are associated with the physiopathology of several NDs including PD [7,8,56]. Glucose is the main source of energy in the brain and the regulation of its metabolism is critical for brain physiology [50]. This is consistent with the observation that *DJ-1β* mutants show a dysregulation of carbohydrates metabolism (Figure 4). Supporting this, a decrease in glucose levels in the iPSC-derived DA neurons mutant for *PARK2* was previously observed [72]. In fact, glucose is the sole substrate that can supply the rapid energy demand of neuronal cells through glycolysis [56,73]. Therefore, an increase in glucose consumption through this pathway to restore ATP levels could explain its reduction in our PD model flies [7,74]. Glycolysis is an ATP-producing pathway that could provide considerable amounts of ATP in a less efficient manner than oxidative phosphorylation to support the acute energy demands in neurons [73,75]. DJ-1 was described as participating in the activity of complex I of ETC binding and stabilizing it [14]. Accordingly, TCA cycle reduction and a higher NADH/NAD^+^ ratio in the PD model flies suggest the existence of an alteration of this complex and mitochondrial dysfunction, which was also observed in a MPTP-induced PD cell model [76]. Thus, our results show that a shift from TCA cycle to glycolysis is produced in PD model flies [7,41,77]. In addition, it was reported that a reduction in Aco activity caused neurotoxicity in mesencephalic rat cultures, due to the loss of its activity as ROS and an iron biosensor [52,78]. Moreover, Aco mutant flies exhibited a reduced locomotor activity, shortened lifespan, and increased cell death in the developing brain, as well as glycolysis and TCA cycle disturbances that led to decreased ATP levels [52]. Some of the phenotypes are similar to those observed in *DJ-1β* mutant flies [7,27]. On the other hand, the reduction in SDH activity leads to the activation of the mammalian target of rapamycin (mTOR) and the sterol regulatory element binding protein (SREBP), which contribute to lipid accumulation in neurons, as observed in several NDs, and produce excitotoxicity, thus participating in PD pathogenesis and development [79,80]. In addition, the finding of reduced malate levels in PD model flies is also noteworthy. Malate is an intermediate of the TCA cycle that is metabolized from fumarate by the fumarate hydratase enzyme. Interestingly, it was shown that the expression of fumarate hydratase was reduced in the DA neurons of the SNpc in the brains of iPD patients [81]. Thus, our results and these observations suggest that a decrease in the activity of both enzymes might be relevant to PD pathogenesis.

Although TCA cycle activity is decreased in *DJ-1β* mutant flies, there is an increase in some pathway intermediates such as fumarate (Figure 4g,h). TCA cycle metabolites can be produced by other pathways, as could be happening with fumarate and the UC [55]. UC is responsible for the excretion of the nitrogen that cannot be used in amino acid metabolism, and is related to the TCA cycle by fumarate, which is transported into the mitochondria, where it can be used as a substrate of TCA cycle [55,56]. The enhanced expression of *arg* and *Argl* in PD model flies could lead to a general increase in UC activity and to higher fumarate levels, as observed in the metabolomic analyses (Figure 4g,h). Supporting these results, an increase in ArgL activity was observed in a zebrafish PD model based on *DJ-1* deficiency [82]. Changes in *arg* expression levels were also reported to have implications in the brain, although its role in this tissue is not yet known [83]. In addition, an enhancement of UC could serve to remove the excess of ammonia caused by the increased amino acid catabolism observed in *DJ-1β* mutant flies, as previously reported in Alzheimer’s disease [83].

## 5. Conclusions

In summary, we demonstrate that loss of *DJ-1β* function leads to several metabolic alterations, such as amino acid metabolism, carbohydrate metabolism, glycolysis, TCA cycle and UC activity in PD model flies. All of them may contribute to PD physiopathology and could constitute possible therapeutic targets for this incurable disease. In addition, disturbances in amino acid levels could be potential biomarkers for both early and later stages of PD. Further studies in other preclinical models of fPD and iPD would be required to confirm the results obtained in *DJ-1β* mutants.

## Figures and Tables

**Table 1:** Most relevant pathways altered in 1-day-old *DJ-1β* mutants compared to control flies.

Pathway	Number of Differential Metabolites/Totals	Raw *p*-Value	*p*-Value FDR Corrected	Impact
beta-Alanine metabolism	2/14	4.21 × 10^−15^	1.43 × 10^−13^	0.28
Histidine metabolism	2/9	7.51 × 10^−13^	8.51 × 10^−12^	0.40
Alanine, aspartate and glutamate metabolism	6/23	2.20 × 10^−12^	1.87 × 10^−11^	0.19
Glyoxylate and dicarboxylate metabolism	6/24	4.07 × 10^−10^	1.54 × 10^−19^	0.17
Phenylalanine, tyrosine and tryptophan biosynthesis	1/4	1.61 × 10^−9^	4.98 × 10^−9^	0.50
Phenylalanine metabolism	1/7	1.61 × 10^−9^	4.98 × 10^−9^	0.38
Glutathione metabolism	1/26	3.84 × 10^−9^	9.33 × 10^−9^	0.09
Glycine, serine and threonine metabolism	3/30	1.35 × 10^−8^	2.90 × 10^−8^	0.33
Pyruvate metabolism	4/22	1.36 × 10^−8^	2.90 × 10^−8^	0.28
Glycerophospholipid metabolism	2/32	1.73 × 10^−8^	3.36 × 10^−8^	0.11
Citrate cycle (TCA cycle)	5/20	9.,20 × 10^−7^	1.56 × 10^−6^	0.24
Glycolysis/Gluconeogenesis	4/26	3.25 × 10^−5^	4.81 × 10^−5^	0.13
Arginine biosynthesis	3/12	7.25 × 10^−5^	1.03 × 10^−4^	0.40
Cysteine and methionine metabolism	2/32	2.29 × 10^−4^	3.11 × 10^−4^	0.14
Taurine and hypotaurine metabolism	1/7	4.72 × 10^−3^	5.48 × 10^−3^	0.20
Arginine and proline metabolism	2/31	4.84 × 10^−3^	5.48 × 10^−3^	0.17

This table presents the most significant pathways (*p*-value FDR corrected < 0.05) with a considerable impact power, which were altered in 1-day-old *DJ-1β* mutant compared to control flies. In particular, the number of differential metabolites/total indicates matched the number of metabolites in the total number of compounds in the pathway; raw *p*-value is the original *p*-value calculated from the enrichment analysis; *p*-value FDR corrected is the *p*-value adjusted using False Discovery Rate; the impact is the pathway impact value calculated from pathway topology analysis.

**Table 2:** Most relevant pathways altered in 15-day-old *DJ-1β* mutants compared to control flies.

Pathway	Number of Differential Metabolites/Totals	Raw *p*-Value	*p*-Value FDR Corrected	Impact
Glyoxylate and dicarboxylate metabolism	6/24	1.03 × 10^−11^	3.50 × 10^−10^	0.17
beta-Alanine metabolism	2/14	8.98 × 10^−11^	1.53 × 10^−9^	0.28
Glycerophospholipid metabolism	2/32	4.74 × 10^−10^	4.15 × 10^−9^	0.11
Citrate cycle (TCA cycle)	5/20	4.89 × 10^−10^	4.15 × 10^−9^	0.24
Taurine and hypotaurine metabolism	1/7	5.08 × 10^−9^	2.88 × 10^−8^	0.20
Histidine metabolism	2/9	2.79 × 10^−8^	1.36 × 10^−7^	0.40
Pyruvate metabolism	4/22	1.88 × 10^−7^	7.09 × 10^−7^	0.28
Alanine, aspartate and glutamate metabolism	6/23	1.33 × 10^−5^	2.66 × 10^−5^	0.19
Glycine, serine and threonine metabolism	3/30	3.65 × 10^−5^	6.20 × 10^−5^	0.33
Arginine biosynthesis	3/12	2.89 × 10^−4^	4.67 × 10^−4^	0.40
Nicotinate and nicotinemide metabolism	1/9	4.03 × 10^−4^	6.23 × 10^−4^	0.37
Tyrosine metabolism	2/33	2.52 × 10^−3^	3.72 × 10^−3^	0.04
Purine metabolism	3/63	7.64 × 10^−3^	9.98 × 10^−3^	0.02
Glycolysis/Gluconeogenesis	4/26	1.76 × 10^−2^	2.21 × 10^−2^	0.13

This table presents the most significant pathways (*p*-value FDR corrected < 0.05) with a considerable impact power, which were altered in 15-day-old *DJ-1β* mutant compared to control flies. In particular, the number of differential metabolites/total indicates matched number of metabolites in the total number of compounds in the pathway; raw *p*-value is the original *p*-value calculated from the enrichment analysis; *p*-value FDR corrected is the *p*-value adjusted using False Discovery Rate; the impact is the pathway impact value calculated from pathway topology analysis.

**Table 3:** Most relevant pathways altered in 15-day-old compared to 1-day-old *DJ-1β* mutants.

Pathway	Number of Differential Metabolites/Totals	Raw *p*-Value	*p*-Value FDR Corrected	Impact
Glycerophospholipid metabolism	2/32	1.85 × 10^−5^	6.29 × 10^−4^	0.11
Alanine, aspartate and glutamate	6/23	1.98 × 10^−4^	3.36 × 10^−3^	0.19
Citrate cycle (TCA cycle)	5/20	4.54 × 10^−3^	2.57 × 10^−2^	0.24
Phenylalanine, tyrosine and tryptophan biosynthesis	1/4	7.60 × 10^−3^	2.87 × 10^−2^	0.50
Phenylalanine metabolism	1/7	7.60 × 10^−3^	2.87 × 10^−2^	0.38

This table presents the most significant pathways (*p*-value FDR corrected < 0.05) with a considerable impact power, which were altered between 1-day-old and 15-day-old *DJ-1β* mutant flies. In particular, the number of differential metabolites/total indicates matched number of metabolites in the total number of compounds in the pathway; raw *p*-value is the original *p*-value calculated from the enrichment analysis; *p*-value FDR corrected is the *p*-value adjusted using False Discovery Rate; the impact is the pathway impact value calculated from pathway topology analysis.

## Data Availability

Not applicable.

## Supplementary Materials

The following are available online at https://www.mdpi.com/article/10.3390/cells11030331/s1, Table S1: NMR data of selected metabolites from extracts of 1-day-old *DJ-1β* mutant and control flies, Table S2: NMR data of selected metabolites from extracts of 15-day-old *DJ-1β* mutant and control flies, Table S3: NMR data of selected metabolites from extracts of 1-day-old and 15-day-old *DJ-1β* mutant flies, Table S4: Results from the pathway enrichment analysis between 1-day-old *DJ-1β* mutant and control flies, Table S5: Selected results from the pathway enrichment analyses in 15-day-old *DJ-1β* mutant and control flies, Table S6: Results from the pathway enrichment analyses in 1-day-old and 15-day-old *DJ-1β* mutant flies.
