# Supplementary material for: Metabolic Alterations in a Drosophila Model of Parkinson’s Disease Based on DJ-1 Deficiency
Source: Cells. 2022 Jan 20;11(3):331. doi: 10.3390/cells11030331 (PMC8834223; doi:10.3390/cells11030331)
Supplement: Supplementary file 1 [file cells-11-00331-s001.zip › Table S1.pdf]

Table S1. NMR data of identified metabolites from extracts of 1-day-old *DJ-1β* mutant and control flies.

Code	Metabolite	NMR region (ppm)		1-day-old control		1-day-old *DJ-1β*		
		Right limit	Left limit	Mean	SEM	Mean	SEM	p-value
Var_150	β-alanine	3.166	3.196	1497.021	10.324	2241.136	53.600	2.345E-09
Var_45	Acetate	1.9	1.947	1254.178	22.332	1161.596	35.112	0.130
Var_254	Acetyl-aspartate	2.7	2.704	4.735	0.140	11.898	0.425	1.181E-10
Var_28	Alanine	1.469	1.505	4138.683	28.840	2749.788	85.309	2.436E-10
Var_132	Anserine	7.078	7.105	425.873	6.873	605.140	23.402	3.273E-05
Var_38	Arginine	1.628	1.659	250.708	6.383	218.632	8.266	4.094E-02
Var_70	Asparagine	2.922	2.928	7.376	0.276	10.465	0.689	0.008
Var_238	ATP/ADP	6.134	6.169	378.967	4.594	500.727	10.853	2.578E-07
Var_261	Citrate	2.514	2.524	28.707	0.576	36.154	1.851	0.013
Var_21	Ethanol	1.17	1.206	1296.167	99.188	1520.882	72.816	0.210
Var_82	Formate	8.455	8.468	41.417	1.579	47.178	2.238	0.151
Var_166	Fructose	3.974	4.034	2339.183	24.596	2667.412	53.791	0.001
Var_111	Fumarate	6.517	6.529	18.963	0.695	23.128	0.762	0.009
Var_220	Glucose	3.709	3.716	698.250	8.449	532.351	8.781	2.402E-09
Var_54	Glutamine	2.114	2.171	1303.499	23.227	1778.299	70.500	1.682E-04
Var_151	Glycerophosphocholine	3.196	3.203	204.725	5.881	119.315	3.520	1.144E-08
Var_184	Glycine	3.557	3.57	848.584	10.408	551.874	11.357	3.371E-12
Var_103	Guanosine	8.002	8.012	20.591	0.405	14.835	0.656	2.657E-05
Var_133	Histidine	7.005	7.019	32.760	0.646	22.541	0.872	1.081E-06
Var_87	Hypoxantine	8.177	8.22	121.817	3.114	141.586	4.964	0.026
Var_7	Isoleucine	1.006	1.027	199.317	3.697	77.223	2.352	1.820E-15
Var_5	Leucine	0.951	0.981	674.070	16.845	264.828	5.178	7.852E-14
Var_8	Leucine (triplete) valine (left singulete)	0.85	0.927	710.251	80.494	543.775	27.838	0.181
Var_195	Lysine	3.045	3.053	156.861	6.285	50.755	4.769	2.990E-09

Var_251	Malate	2.633	2.642	47.312	1.895	12.998	0.677	3.613E-11
Var_40	Methionine	1.703	1.769	687.468	8.824	592.802	12.880	2.990E-04
Var_62	Methionine-sulfoxide	2.745	2.77	1572.816	63.483	472.262	46.353	1.448E-09
Var_256	N-acetyl aspartate	2.709	2.716	15.632	0.502	24.953	0.801	5.356E-07
Var_149	NAD+	4.478	4.502	59.812	1.621	60.424	1.586	0.850
Var_165	NADH	4.203	4.239	137.540	10.262	232.759	9.099	6.565E-05
Var_273	O-phosphocholine	3.219	3.242	5324.258	56.353	7041.547	245.812	8.252E-05
Var_117	Phenylalanine	7.415	7.427	36.403	0.599	22.365	0.323	8.548E-13
Var_121	Phenylalanine (sing duplete)	7.375	7.387	24.617	0.436	17.137	0.632	6.416E-07
Var_162	Phosphocholine	4.155	4.19	664.400	11.139	905.857	48.016	0.002
Var_47	Proline	1.961	1.966	9.090	0.200	8.043	0.224	0.022
Var_230	Pyruvate	2.368	2.382	944.063	45.551	651.117	38.027	0.002
Var_59	Succinate	2.398	2.417	746.371	22.023	663.889	24.254	0.089
Var_279	Threonine	1.334	1.35	593.651	11.041	329.348	5.580	4.256E-13
Var_309	Trehalose	5.185	5.214	225.223	13.010	1676.976	162.136	2.373E-06
Var_99	Tryptophane	7.745	7.752	11.078	0.224	9.827	0.500	0.121
Var_9	Valine	0.928	0.934	45.202	1.676	26.562	0.438	1.341E-07

Note: For each peak we indicate the **code** (variable number for identified metabolites), the integration range (NMR region), the mean of the twelve experimental replicates, and the standard error of the mean (SEM). In all cases we also indicate the statistical significance value (p-value) of the comparison to the corresponding control. All means are highlighted in blue, significant differences (*P*<0.05) are highlighted in red.
