# Supplementary material for: Metabolic Alterations in a Drosophila Model of Parkinson’s Disease Based on DJ-1 Deficiency
Source: Cells. 2022 Jan 20;11(3):331. doi: 10.3390/cells11030331 (PMC8834223; doi:10.3390/cells11030331)
Supplement: Supplementary file 1 [file cells-11-00331-s001.zip › Table S2.pdf]

Table S2. NMR data of identified metabolites from extracts of 15-day-old *DJ-1β* mutant and control flies.

Code	Metabolite	NMR region (ppm)		15-day-old control		15-day-old *DJ-1β*		
		Right limit	Left limit	Mean	SEM	Mean	SEM	p-value
Var_150	β-alanine	3.166	3.196	1453.841	17.053	2139.360	35.987	3.022E-11
Var_45	Acetate	1.9	1.947	1295.232	26.367	1147.969	21.278	0.006
Var_254	Acetyl-aspartate	2.7	2.704	5.276	0.294	9.986	0.321	1.211E-07
Var_28	Alanine	1.469	1.505	5017.420	97.368	3767.206	63.851	1.391E-07
Var_132	Anserine	7.078	7.105	360.865	5.271	456.221	10.595	9.910E-06
Var_38	Arginine	1.628	1.659	310.082	8.033	242.824	7.176	2.188E-04
Var_70	Asparagine	2.922	2.928	7.285	0.384	13.328	0.543	1.813E-06
Var_238	ATP/ADP	6.134	6.169	403.078	6.357	451.620	6.684	0.001
Var_261	Citrate	2.514	2.524	27.319	0.669	32.061	0.755	0.003
Var_21	Ethanol	1.17	1.206	931.541	102.702	1403.548	68.815	0.013
Var_82	Formate	8.455	8.468	50.902	3.323	50.819	2.866	0.989
Var_166	Fructose	3.974	4.034	2365.192	31.149	2223.779	46.507	0.088
Var_111	Fumarate	6.517	6.529	24.352	0.674	29.479	0.604	0.001
Var_220	Glucose	3.709	3.716	736.234	10.057	679.685	23.351	0.130
Var_54	Glutamine	2.114	2.171	1700.391	22.223	1716.533	43.923	0.819
Var_151	Glycerophosphocholine	3.196	3.203	248.304	5.412	110.621	5.423	1.313E-11
Var_184	Glycine	3.557	3.57	959.053	11.671	618.891	10.326	2.760E-13
Var_103	Guanosine	8.002	8.012	20.388	0.563	16.361	0.654	0.003
Var_133	Histidine	7.005	7.019	36.499	0.772	22.519	1.080	1.902E-07
Var_87	Hypoxantine	8.177	8.22	135.131	2.814	145.700	4.348	0.163
Var_7	Isoleucine	1.006	1.027	189.573	4.661	98.821	2.161	1.833E-11
Var_5	Leucine	0.951	0.981	671.758	23.862	341.061	10.773	9.048E-09
Var_8	Leucine (triplete) valine (left singulete)	0.85	0.927	716.896	80.591	593.052	20.433	0.304
Var_195	Lysine	3.045	3.053	114.063	3.486	42.049	3.532	7.535E-10

Var_251	Malate	2.633	2.642	38.200	0.765	13.489	0.461	2.111E-15
Var_40	Methionine	1.703	1.769	775.848	14.961	591.805	7.173	8.178E-08
Var_62	Methionine-sulfoxide	2.745	2.77	1186.373	28.449	398.954	36.364	3.616E-11
Var_256	N-acetyl aspartate	2.709	2.716	13.377	0.397	23.790	0.507	9.923E-11
Var_149	NAD+	4.478	4.502	57.856	1.284	68.796	1.927	0.003
Var_165	NADH	4.203	4.239	112.346	3.955	189.451	5.430	4.643E-08
Var_273	O-phosphocholine	3.219	3.242	4407.078	47.757	4587.715	83.914	0.199
Var_117	Phenylalanine	7.415	7.427	34.549	1.269	26.816	0.482	0.001
Var_121	Phenylalanine (sing duplete)	7.375	7.387	24.758	0.970	17.897	0.858	0.001
Var_162	Phosphocholine	4.155	4.19	525.027	6.400	527.608	13.014	0.901
Var_47	Proline	1.961	1.966	8.878	0.394	7.916	0.205	0.140
Var_230	Pyruvate	2.368	2.382	764.206	75.314	624.574	20.559	0.219
Var_59	Succinate	2.398	2.417	703.303	19.558	567.353	14.091	0.001
Var_279	Threonine	1.334	1.35	526.765	14.004	324.438	6.417	4.553E-09
Var_309	Trehalose	5.185	5.214	286.752	16.941	1222.110	121.029	1.954E-05
Var_99	Tryptophane	7.745	7.752	12.467	0.427	10.620	0.344	0.026
Var_9	Valine	0.928	0.934	46.385	1.983	30.615	0.801	3.145E-05

Note: For each peak we indicate the **code** (variable number for identified metabolites), the integration range (NMR region), the mean of the twelve experimental replicates, and the standard error of the mean (SEM). In all cases we also indicate the statistical significance value (p-value) of the comparison to the corresponding control. All means are highlighted in blue, significant differences (*P*<0.05) are highlighted in red.
