# Supplementary material for: Metabolic Alterations in a Drosophila Model of Parkinson’s Disease Based on DJ-1 Deficiency
Source: Cells. 2022 Jan 20;11(3):331. doi: 10.3390/cells11030331 (PMC8834223; doi:10.3390/cells11030331)
Supplement: Supplementary file 1 [file cells-11-00331-s001.zip › Table S3.pdf]

Table S3. NMR data of identified metabolites from extracts of 1-day-old and 15-day-old *DJ-1β* mutant flies

Code	Metabolite	NMR region (ppm)		1-day-old *DJ-1β* mutant		15-day-old *DJ-1β* mutant		
		Right limit	Left limit	Mean	SEM	Mean	SEM	p-value
Var_150	β-alanine	3.166	3.196	2241.136	53.600	2139.360	35.987	0.277
Var_45	Acetate	1.9	1.947	1161.596	35.112	1147.969	21.278	0.817
Var_254	Acetyl-aspartate	2.7	2.704	11.898	0.425	9.986	0.321	0.019
Var_28	Alanine	1.469	1.505	2749.788	85.309	3767.206	63.851	8.738E-07
Var_132	Anserine	7.078	7.105	605.140	23.402	456.221	10.595	4.736E-04
Var_38	Arginine	1.628	1.659	218.632	8.266	242.824	7.176	0.132
Var_70	Asparagine	2.922	2.928	10.465	0.689	13.328	0.543	0.031
Var_238	ATP/ADP	6.134	6.169	500.727	10.853	451.620	6.684	0.012
Var_261	Citrate	2.514	2.524	36.154	1.851	32.061	0.755	0.162
Var_21	Ethanol	1.17	1.206	1520.882	72.816	1403.548	68.815	0.416
Var_82	Formate	8.455	8.468	47.178	2.238	50.819	2.866	0.486
Var_166	Fructose	3.974	4.034	2667.412	53.791	2223.779	46.507	2.208E-04
Var_111	Fumarate	6.517	6.529	23.128	0.762	29.479	0.604	1.328E-04
Var_220	Glucose	3.709	3.716	532.351	8.781	679.685	23.351	3.926E-04
Var_54	Glutamine	2.114	2.171	1778.299	70.500	1716.533	43.923	0.604
Var_151	Glycerophosphocholine	3.196	3.203	119.315	3.520	110.621	5.423	0.352
Var_184	Glycine	3.557	3.57	551.874	11.357	618.891	10.326	0.005
Var_103	Guanosine	8.002	8.012	14.835	0.656	16.361	0.654	0.256
Var_133	Histidine	7.005	7.019	22.541	0.872	22.519	1.080	0.991
Var_87	Hypoxantine	8.177	8.22	141.586	4.964	145.700	4.348	0.664
Var_7	Isoleucine	1.006	1.027	77.223	2.352	98.821	2.161	8.950E-05
Var_5	Leucine	0.951	0.981	264.828	5.178	341.061	10.773	1.736E-04
Var_8	Leucine (triplete) valine (left singulete)	0.85	0.927	543.775	27.838	593.052	20.433	0.324
Var_195	Lysine	3.045	3.053	50.755	4.769	42.049	3.532	0.311

Var_251	Malate	2.633	2.642	12.998	0.677	13.489	0.461	0.676
Var_40	Methionine	1.703	1.769	592.802	12.880	591.805	7.173	0.962
Var_62	Methionine-sulfoxide	2.745	2.77	472.262	46.353	398.954	36.364	0.388
Var_256	N-acetyl aspartate	2.709	2.716	24.953	0.801	23.790	0.507	0.395
Var_149	NAD+	4.478	4.502	60.424	1.586	68.796	1.927	0.027
Var_165	NADH	4.203	4.239	232.759	9.099	189.451	5.430	0.008
Var_273	O-phosphocholine	3.219	3.242	7041.547	245.812	4587.715	83.914	1.025E-06
Var_117	Phenylalanine	7.415	7.427	22.365	0.323	26.816	0.482	1.907E-05
Var_121	Phenylalanine (sing duplete)	7.375	7.387	17.137	0.632	17.897	0.858	0.619
Var_162	Phosphocholine	4.155	4.19	905.857	48.016	527.608	13.014	2.128E-05
Var_47	Proline	1.961	1.966	8.043	0.224	7.916	0.205	0.772
Var_230	Pyruvate	2.368	2.382	651.117	38.027	624.574	20.559	0.668
Var_59	Succinate	2.398	2.417	663.889	24.254	567.353	14.091	0.024
Var_279	Threonine	1.334	1.35	329.348	5.580	324.438	6.417	0.687
Var_309	Trehalose	5.185	5.214	1676.976	162.136	1222.110	121.029	0.126
Var_99	Tryptophane	7.745	7.752	9.827	0.500	10.620	0.344	0.366
Var_9	Valine	0.928	0.934	26.562	0.438	30.615	0.801	0.005

Note: For each peak we indicate the **code** (variable number for identified metabolites), the integration range (NMR region), the mean of the twelve experimental replicates, and the standard error of the mean (SEM). In all cases we also indicate the statistical significance value (p-value) of the comparison to the corresponding control. All means are highlighted in blue, significant differences (P<0.05) are highlighted in red.
