# Supplementary material for: Metabolic Alterations in a Drosophila Model of Parkinson’s Disease Based on DJ-1 Deficiency
Source: Cells. 2022 Jan 20;11(3):331. doi: 10.3390/cells11030331 (PMC8834223; doi:10.3390/cells11030331)
Supplement: Supplementary file 1 [file cells-11-00331-s001.zip › Table S4.pdf]

Table S4. . Results from the pathway enrichment analysis between 1-day-old *DJ-1β* mutants and control flies.

**Pathway**	**Number of differential metabolites/totals**	**Raw p-value**	**p-value FDR corrected**	**Impact**
beta-Alanine metabolism	2/14	4,21E-15	1,43E-13	0,28
Pantothenate and CoA biosynthesis	2/18	4,82E-13	8,20E-12	0,00
Histidine metabolism	2/9	7,51E-13	8,51E-12	0,40
Alanine, aspartate and glutamate metabolism	6/23	2,20E-12	1,87E-11	0,19
Valine, leucine and isoleucine biosynthesis	4/8	6,40E-12	4,35E-11	0,00
Valine, leucine and isoleucine degradation	3/38	1,99E-11	1,13E-10	0,00
Aminoacyl-tRNA biosynthesis	15/48	4,03E-11	1,96E-10	0,00
Starch and sucrose metabolism	1/14	1,21E-10	5,14E-10	0,01
Glyoxylate and dicarboxylate metabolism	6/24	4,07E-10	1,54E-09	0,17
Phenylalanine, tyrosine and tryptophan biosynthesis	1/4	1,61E-09	4,98E-09	0,50
Phenylalanine metabolism	1/7	1,61E-09	4,98E-09	0,38
Propanoate metabolism	2/21	3,26E-09	9,25E-09	0,00
Glutathione metabolism	1/26	3,84E-09	9,33E-09	0,09
Porphyrin and chorophyll metabolism	1/24	3,84E-09	9,33E-09	0,00
Glycine, serine and threonine metabolism	3/30	1,35E-08	2,90E-08	0,33
Pyruvate metabolism	4/22	1,36E-08	2,90E-08	0,28
Glycerophospholipid metabolism	2/32	1,73E-08	3,36E-08	0,11
Lysine degradation	1/21	1,15E-07	2,06E-07	0,00
Biotin metabolism	1/10	1,15E-07	2,06E-07	0,00
Citrate cycle (TCA cycle)	5/20	9,20E-07	1,56E-06	0,24
Pyrimidine metabolism	2/40	2,93E-06	4,74E-06	0,00
Purine metabolism	3/63	4,20E-06	6,50E-06	0,02
Glycolysis/Gluconeogenesis	4/26	3,25E-05	4,81E-05	0,13
Arginine biosynthesis	3/12	7,25E-05	1,03E-04	0,40
Cysteine and methionine metabolism	2/32	2,29E-04	3,11E-04	0,14

D-Glutamine and D-glutamate metabolism	1/5	4,54E-04	5,72E-04	0,00
Nitrogen metabolism	1/5	4,54E-04	5,72E-04	0,00
Tyrosine metabolism	2/33	5,15E-04	6,26E-04	0,04
Taurine and hypotaurine metabolism	1/7	4,72E-03	5,48E-03	0,20
Arginine and proline metabolism	2/31	4,84E-03	5,48E-03	0,17
Amino sugar and nucleotide sugar metabolism	1/34	3,12E-02	3,42E-02	0,00
Tryptophan metabolism	1/30	1,07E-01	1,14E-01	0,21
Butanoate metabolism	1/14	1,11E-01	1,14E-01	0,00
Nicotinate and nicotinamide metabolism	1/9	9,52E-01	9,52E-01	0,37
D-Glutamine and D-glutamate metabolism	1/5	4,54E-04	5,72E-04	0,00

Note: **Number of differential metabolites/total** indicates matched number of metabolites in the total number of compounds in the pathway; **raw p-value** is the original p-value calculated from the enrichment analysis; **p-value FDR corrected** is the p-value adjusted using False Discovery Rate; the **impact** is the pathway impact value calculated from pathway topology analysis. Significant differences (p-value FDR corrected<0.05) are highlighted in blue; high impact scores among significant pathways are highlighted in yellow.
