# Supplementary material for: Metabolic Alterations in a Drosophila Model of Parkinson’s Disease Based on DJ-1 Deficiency
Source: Cells. 2022 Jan 20;11(3):331. doi: 10.3390/cells11030331 (PMC8834223; doi:10.3390/cells11030331)
Supplement: Supplementary file 1 [file cells-11-00331-s001.zip › Table S5.pdf]

Table S5. Selected results from the pathway enrichment analyses in 15-day-old *DJ-1β* mutants and control flies.

Pathway	Number of differential metabolites/totals	Raw p-value	p-value FDR corrected	Impact
Glyoxylate and dicarboxylate metabolism	6/24	1,03E-11	3,50E-10	0,17
beta-Alanine metabolism	2/14	8,98E-11	1,53E-09	0,28
Glycerophospholipid metabolism	2/32	4,74E-10	4,15E-09	0,11
Citrate cycle (TCA cycle)	5/20	4,89E-10	4,15E-09	0,24
Pantothenate and CoA biosynthesis	2/18	2,63E-09	1,79E-08	0,00
Taurine and hypotaurine metabolism	1/7	5,08E-09	2,88E-08	0,20
Histidine metabolism	2/9	2,79E-08	1,36E-07	0,40
Propanoate metabolism	2/21	1,15E-07	4,89E-07	0,00
Pyruvate metabolism	4/22	1,88E-07	7,09E-07	0,28
Lysine degradation	1/21	7,13E-07	2,20E-06	0,00
Biotin metabolism	1/10	7,13E-07	2,20E-06	0,00
Valine, leucine and isoleucine biosynthesis	4/8	1,24E-06	3,52E-06	0,00
Starch and sucrose metabolism	1/14	1,52E-06	3,98E-06	0,01
Aminoacyl-tRNA biosynthesis	15/48	2,13E-06	5,17E-06	0,00
Valine, leucine and isoleucine degradation	3/38	3,20E-06	7,25E-06	0,00
Pyrimidine metabolism	2/40	6,35E-06	1,35E-05	0,00
Alanine, aspartate and glutamate metabolism	6/23	1,33E-05	2,66E-05	0,19
Glutathione metabolism	1/26	1,74E-05	3,11E-05	0,09
Porphyrin and chlorophyll metabolism	1/24	1,74E-05	3,11E-05	0,00
Glycine, serine and threonine metabolism	3/30	3,65E-05	6,20E-05	0,33
Arginine biosynthesis	3/12	2,89E-04	4,67E-04	0,40
Nicotinate and nicotinemide metabolism	1/9	4,03E-04	6,23E-04	0,37
Tyrosine metabolism	2/33	2,52E-03	3,72E-03	0,04
D-Glutamine and D-Glutamate metabolism	1/5	7,31E-03	9,94E-03	0,00
Nitrogen metabolism	1/5	7,31E-03	9,94E-03	0,00

Purine metabolism	3/63	7,64E-03	9,98E-03	0,02
Glycolysis/Gluconeogenesis	4/26	1,76E-02	2,21E-02	0,13
Amino sugar and nucleotide sugar metabolism	1/34	9,32E-02	1,13E-01	0,00
Cysteine and methionine metabolism	2/32	1,86E-01	2,18E-01	0,14
Arginine and proline metabolism	2/31	2,56E-01	2,90E-01	0,17
Phenylalanine, tyrosine and tryptophan biosynthesis	1/4	3,06E-01	3,25E-01	0,50
Phenylalanine metabolism	1/7	3,06E-01	3,25E-01	0,38
Butanoate metabolism	1/14	4,49E-01	4,63E-01	0,00
Tryptophan metabolism	1/30	9,60E-01	9,60E-01	0,21
Glyoxylate and dicarboxylate metabolism	6/24	1,03E-11	3,50E-10	0,17

Note: In particular, **number of differential metabolites/total** indicates matched number of metabolites in the total number of compounds in the pathway; **raw p-value** is the original p-value calculated from the enrichment analysis; **p-value FDR corrected** is the p-value adjusted using False Discovery Rate; the **impact** is the pathway impact value calculated from pathway topology analysis. Significant differences (p-value FDR corrected<0.05) are highlighted in blue; high impact scores among significant pathways are highlighted in yellow.
