# Supplementary material for: Metabolic Alterations in a Drosophila Model of Parkinson’s Disease Based on DJ-1 Deficiency
Source: Cells. 2022 Jan 20;11(3):331. doi: 10.3390/cells11030331 (PMC8834223; doi:10.3390/cells11030331)
Supplement: Supplementary file 1 [file cells-11-00331-s001.zip › Table S6.pdf]

Table S6. Results from the pathway enrichment analyses in 1-day-old and 15-day-old *DJ-1β* mutant flies.

Pathway	Number of differential metabolites/totals	Raw p-value	p-value FDR corrected	Impact
Glycerophospholipid metabolism	2/32	1,85E-05	6,29E-04	0,11
Alanine, aspartate and glutamate	6/23	1,98E-04	3,36E-03	0,19
Valine, leucine and isoleucine degradation	3/38	2,27E-03	1,93E-02	0,00
Amino sugar and nucleotide sugar metabolism	1/34	2,27E-03	1,93E-02	0,00
Valine, leucine and isoleucine biosynthesis	4/8	3,28E-03	2,23E-02	0,00
Citrate cycle (TCA cycle)	5/20	4,54E-03	2,57E-02	0,24
Aminoacyl-tRNA biosynthesis	15/48	6,99E-03	2,87E-02	0,00
Phenylalanine, tyrosine and tryptophan biosynthesis	1/4	7,60E-03	2,87E-02	0,50
Phenylalanine metabolism	1/7	7,60E-03	2,87E-02	0,38
Histidine metabolism	2/9	1,75E-02	5,83E-02	0,40
Tyrosine metabolism	2/33	1,89E-02	5,83E-02	0,04
Butanoate metabolism	1/14	2,23E-02	6,32E-02	0,00
Arginine biosynthesis	3/12	2,82E-02	7,38E-02	0,40
Glutathione metabolism	1/26	4,62E-02	1,05E-01	0,09
Porphyrin and chlorophyll metabolism	1/24	4,62E-02	1,05E-01	0,00
Propanoate metabolism	2/21	6,00E-02	1,27E-01	0,00
Pyruvate metabolism	4/22	1,05E-01	1,99E-01	0,28
Starch and sucrose metabolism	1/14	1,08E-01	1,99E-01	0,01
Glycolysis/Gluconeogenesis	4/26	1,11E-01	1,99E-01	0,13
Nicotinate and nicotinamide metabolism	1/9	1,25E-01	2,11E-01	0,37
Pantothenate and CoA biosynthesis	2/18	1,30E-01	2,11E-01	0,00
Glyoxylate and dicarboxylate metabolism	6/24	1,43E-01	2,20E-01	0,17
Glycine, serine and threonine metabolism	3/30	2,20E-01	3,26E-01	0,33
Arginine and proline metabolism	2/31	2,50E-01	3,54E-01	0,17
Lysine degradation	1/21	3,36E-01	4,40E-01	0,00

Biotin metabolism	1/10	3,36E-01	4,40E-01	0,00
Tryptophan metabolism	1/30	4,21E-01	5,20E-01	0,21
Taurine and hypotaurine metabolism	1/7	4,30E-01	5,20E-01	0,20
Pyrimidine metabolism	2/40	4,43E-01	5,20E-01	0,00
beta-Alanine metabolism	2/14	5,36E-01	6,07E-01	0,28
D-Glutamine and D-glutamate metabolism	1/5	6,16E-01	6,55E-01	0,00
Nitrogen metabolism	1/5	6,16E-01	6,55E-01	0,00
Purine metabolism	3/63	7,08E-01	7,30E-01	0,02
Cysteine and methionine metabolism	2/32	9,08E-01	9,08E-01	0,14
Glycerophospholipid metabolism	2/32	1,85E-05	6,29E-04	0,11

Note: In particular, **number of differential metabolites/total** indicates matched number of metabolites in the total number of compounds in the pathway; **raw p-value** is the original p-value calculated from the enrichment analysis; **p-value FDR corrected** is the p-value adjusted using False Discovery Rate; the **impact** is the pathway impact value calculated from pathway topology analysis. Significant differences (p-value FDR corrected<0.05) are highlighted in blue; high impact scores among significant pathways are highlighted in yellow.
